# Sensory modality and information domain contribute jointly to dual-task interference between working memory and perceptual processing

**DOI:** 10.1162/imag_a_00130

**Published:** 2024-04-08

**Authors:** Justin T. Fleming, J. Michelle Njoroge, Abigail L. Noyce, Tyler K. Perrachione, Barbara G. Shinn-Cunningham

**Affiliations:** Department of Speech-Language-Hearing Sciences, University of Minnesota, Minneapolis, MN, United States; Department of Speech, Language, and Hearing Sciences, Boston University, Boston, MA, United States; Neuroscience Institute, Carnegie Mellon University, Pittsburgh, PA, United States

**Keywords:** multisensory, spatial, temporal, working memory, pupillometry, electroencephalography (EEG)

## Abstract

Making sense of our environment requires us to extract temporal and spatial information from multiple sensory modalities, particularly audition and vision. Often, we must hold this sensory information in working memory (WM) to guide future actions, while simultaneously processing new sensory inputs as they arise. However, these processes of WM maintenance and perceptual processing can interfere with one another when the tasks rely on similar cognitive resources. fMRI studies have uncovered attention and WM networks that are specialized for either auditory-temporal or visual-spatial processing; the functional specialization of these networks makes specific predictions about patterns of interference between perceptual processing and WM. Specifically, we hypothesized that dual-task interference should increase when the tasks share a common sensory modality, a common information domain (temporal vs. spatial processing), or both. To test these predictions, we asked participants to store temporal or spatial information about auditory or visual stimuli in WM. On some trials, participants also performed an intervening auditory task, which was either temporal or spatial, during WM retention. Errors on WM recall and perceptual judgment tasks both generally increased when the tasks relied on shared modality- and domain-biased resources, with maximal interference when both tasks were auditory-temporal. Pupil dilations were also larger and started earlier when both tasks were auditory-temporal, indicating an increase in cognitive effort to overcome the interference. Event-related potentials (ERPs) and alpha-band oscillatory activity revealed neural signatures of domain-based interference even when the tasks were presented in different sensory modalities, when behavioral differences were masked by ceiling effects. These results demonstrate that sensory modality and information domain jointly affect how task information is represented in WM, consistent with past work demonstrating how tasks engage complementary auditory-temporal and visual-spatial cognitive control networks.

## 1 Introduction

Navigating everyday environments requires us to process spatial and temporal information from multiple sensory modalities—particularly vision and audition—and store this information in working memory (WM) to guide future actions. For instance, when crossing a busy street, we first look in one direction, sampling the spatial positions of vehicles over time to determine their direction and speed of movement. This information is then stored in WM while we look the other way to determine the paths of vehicles approaching from that direction. To determine when it is safe to cross, we must maintain the temporal and spatial information stored in WM while simultaneously processing new sensory inputs.

A challenge of maintaining information in WM while processing additional sensory inputs is that these cognitive processes have the potential to interfere with one another. Such dual-task interference is generally strongest when the tasks feature similar stimuli or information processing demands, a pattern at the center of “multiple resource theory” (Navon & Gopher, 1979;Nickerson, 1980;Wickens, 2002). Multiple resource theory states that, in addition to a shared “pool” of general executive or attentional resources, individual tasks draw on specific “satellite” resource pools. When two tasks draw on the same pool of cognitive resources, competition for those limited resources leads to greater behavioral interference between tasks. Conversely, two tasks that predominantly rely on separate pools of cognitive resources should interfere relatively little with one another.

Using this framework, dual-task studies have identified several stimulus attributes and task demands that increase interference when they are shared between tasks. One dimension that reliably modulates interference is sensory modality; perceptual processing interferes more with WM recall when both tasks are auditory, visual, or somatosensory, as compared to when tasks are presented in different sensory modalities (Morrison et al., 2015;Scerra & Brill, 2012). Similarly, auditory and visual discrimination thresholds (for pitch and contrast, respectively) are unaffected by concurrent distractor stimuli in the opposite modality, but are made worse by distractor stimuli in the same modality (Alais et al., 2006). Dual-task paradigms have also identified resource bottlenecks when two tasks rely on the same type of information processing. This literature has largely focused on contrasting verbal/linguistic tasks with other types of task demand (often spatial processing tasks), with results consistently indicating maximal interference when both tasks are linguistic in nature (Wickens, 2008). As a specific example, interference in a word-color Stroop task increases when WM is loaded with verbal information, but not when WM is loaded with unrelated spatial information (Kim et al., 2005).

Patterns of dual-task interference based on sensory modality are compatible with the functional organization of human attention and WM networks. fMRI studies have identified distinct regions within the human lateral frontal cortex (LFC) that are preferentially driven by either auditory or visual information during attention and WM tasks (Braga et al., 2017;Mayer et al., 2016;Michalka et al., 2015;Noyce et al., 2017). Activity in these LFC regions is closely linked to activity in posterior brain areas responsible for auditory or visual attention, forming two networks tuned to processing either auditory or visual information and storing it in WM (Michalka et al., 2015;Tobyne et al., 2017). If these sensory-biased networks are considered as “resources” in a multiple resource theory framework, then concurrent tasks in the same sensory modality should both rely on a single network and therefore interfere with one another. Conversely, tasks in different sensory modalities should be able to leverage both networks, reducing resource bottlenecks.

The organization of these attention and WM networks also predicts interference based on a particular contrast in information domain: temporal versus spatial processing. Although each LFC network specializes in processing information from one sensory modality, these networks are also engaged differentially depending on whether the task is temporal or spatial in nature. Vision and audition have complementary strengths for spatial and temporal processing, respectively. Starting at the retina, visual representations are inherently spatial, and neural maps of space are found throughout the visual processing pathway (Silver & Kastner, 2009;Stensaas et al., 1974;Swisher et al., 2007). In contrast, auditory spatial information must be computed by comparing the signals reaching the two ears. The peripheral auditory system instead excels at representing temporal information (Dynes & Delgutte, 1992), leading to much better perceptual sensitivity for temporal information in audition than vision (Rammsayer, 2014). Patterns of LFC network recruitment reflect these complementary specializations. Specifically, auditory tasks that involve*spatial*information processing recruit the*visual*-biased LFC network, while visual tasks that involve*temporal*processing recruit the*auditory*-biased LFC network (Michalka et al., 2015). Thus, information from*either*sensory modality seems to be processed by the network specialized for representing a particular information domain.

The activity patterns of these LFC networks lead to previously untested, mechanism-grounded predictions about how perceptual and WM tasks should interfere on the basis of sensory modality and information domain. First, the fact that these brain networks are tuned to temporal or spatial processing suggests that this distinction in information domain should modulate dual-task interference. Second, in a dual-task paradigm that engages the LFC networks, interference should depend on the*interaction*of sensory modality and information domain between the two tasks. For example, an auditory perceptual task should interfere more with auditory information held in WM than visual information, and this interference should be magnified if the auditory perceptual task is also temporal (fully relying on the auditory LFC network) as compared to spatial. Whereas most dual-task studies focus on a single stimulus or task dimension of interest, a major goal of the current project was to test these interactive interference effects predicted by the organization of attention and WM brain networks.

To examine these hypotheses, we tested interference effects using a novel dual-task paradigm featuring WM and “Intervening” tasks. The WM task required participants to remember either temporal or spatial information about a set of non-linguistic auditory or visual stimuli. The WM task conditions were derived from tasks used to recruit the complementary LFC networks in fMRI studies (Michalka et al., 2015), giving us high confidence that these networks would be engaged by the WM component of our dual-task design. The Intervening task, which was presented during WM retention, required participants to make an immediate perceptual judgment about auditory stimuli. We hypothesized that dual-task interference would be high when the WM task was auditory-temporal, because in this case, both tasks would rely exclusively on the auditory-biased WM and attention network. When the WM task was instead auditory-*spatial*, we expected that WM maintenance would recruit the visual-biased network, reducing the bottleneck on auditory-biased resources and thereby reducing interference from the auditory Intervening task. In the visual WM conditions, we expected interference to be generally low as both control networks could be leveraged in parallel.

The auditory Intervening task also had a temporal and a spatial variant, allowing us to examine additional interactions between the information domains of the WM and Intervening tasks (i.e., would interference increase if both tasks were temporal or spatial?). The Intervening task was designed to stress perceptual processing while having minimal memory demands, and therefore it required only immediate judgments about sound timing or locations. Due to the limited demands on spatial memory, we expected that the auditory-spatial Intervening task might not recruit the visual-biased network as robustly as the auditory-spatial WM task. Thus, we expected that the information domain of the Intervening task might have relatively subtle effects on dual-task interference, whereas the domain of the WM task would more robustly modulate interference.

In addition to behavioral measures of interference, we also examined how autonomic and neural signatures of interference were affected by modality and domain similarity between the WM and Intervening tasks. Pupillometry and electroencephalography (EEG) data were continuously recorded throughout each dual-task trial. Pupillometry serves as a sensitive psychophysiological index of the cognitive effort required to achieve a certain performance level, even when there are not measurable differences in behavioral outcomes (Causse et al., 2016;Gilzenrat et al., 2012;Murphy et al., 2011;Winn et al., 2015). For instance, speech processing evokes larger pupil dilations for participants with hearing loss than normal-hearing controls, even in favorable listening conditions where both groups achieve ceiling performance at speech recognition (Ohlenforst et al., 2017). In the current study, we included pupillometry for its ability to detect subtle interference effects that may have been masked in the behavioral data. As a time-series measure, pupillometry can also provide insights into*when*participants deployed effort over the course of a trial; here, we leverage this strength to shed light on the timing of effort as participants prepared for upcoming high-interference dual-task conditions.

The EEG data were included so we could test two hypotheses about the neural underpinnings of behavioral interference effects in our dual-task paradigm. First, we analyzed event-related potentials (ERPs) to test whether pre-loading the auditory- or the visual-biased WM and attention network (using the WM task) would differentially affect early sensory responses to auditory stimuli in the Intervening task. We focused on these Intervening task ERPs for both theoretical and technical reasons. The Intervening task phase was of theoretical interest because it was the window in which participants were simultaneously performing a perceptual task while also maintaining the integrity of information held in WM. From a technical perspective, focusing our analysis on the Intervening task also allowed us to compare ERPs elicited by physically identical auditory stimuli, differing only in their modality and domain relationship to the information held in WM. Early components of the auditory ERP—in particular the N1 and P2 components—are known to be modulated by selective attention (Hillyard et al., 1973;Woldorff et al., 1993). Previous dual-task studies have shown that performing a simultaneous secondary visual task reduces the amplitudes of N1 ERP components elicited by auditory stimuli (Parasuraman, 1985;Singhal et al., 2002). We hypothesized that the magnitude of such N1 reductions might depend on interactions between the sensory modality and information domain of the two tasks. In particular, pre-loading the auditory-biased network with an auditory WM task might create a bottleneck on attentional resources needed for the auditory Intervening task, leading to reduced ERP amplitudes.

The second hypothesis we examined with EEG was that the Intervening task might interrupt WM maintenance, and that this form of interference would also be stronger when the two tasks shared a common sensory modality and/or information domain. To test this, we leveraged an established electrocortical signature of WM maintenance: oscillatory power in the alpha (8–12 Hz) band (Klimesch, 1999). At least one previous study has shown interruption of this ongoing alpha activity during WM maintenance by distracting stimuli (Hakim et al., 2020;Mishra et al., 2013). We examined whether the Intervening task would cause a similar disruption of alpha activity in our paradigm, and whether this disruption would be more severe when the WM and Intervening tasks relied on shared network resources.

Across the behavioral, pupillometry, and EEG measures, we expected that interference would be heightened when the two tasks relied on similar WM and attention networks. Broadly, this should map onto the most interference when the tasks are presented in the same sensory modality and require processing information in the same domain, with interference decreasing when modality, domain, or both differ between tasks. Behaviorally, we expected interference to manifest as a bidirectional performance decrement, with both WM and Intervening task errors increasing in the high-interference conditions. In the pupil data, we expected to see larger pupil dilations during (and perhaps in anticipation of) Intervening task performance when WM was loaded with similar information, indicating elevated cognitive effort to overcome the interference. Finally, in conditions with modality and/or domain overlap between tasks, we expected that ERPs elicited by Intervening task stimuli would have smaller amplitudes and that alpha power during WM maintenance would be interrupted to a greater extent.

## 2 Materials and Methods

### 2.1 Participants

Twenty-three healthy young adults completed all experimental procedures. Data from three of these participants were removed due to excessive noise in the pupillometry or EEG recordings, yielding a final sample of*N*= 20 (13 female; mean age 20.9 years; range 18 to 28 years). This recruitment target came from a power analysis informed by behavioral pilot data (*N*= 6), which indicated that 15 participants would be required to detect performance differences in our auditory perceptual tasks based on the type of information held in WM, assuming a moderate effect size (0.5) and a alpha level of 0.05. To account for potential additional variability in the EEG and pupillometry data, this recruitment target was increased to 20.

One participant was excluded from only the time-frequency analyses due to anomalous high-frequency noise (increasing above 30 Hz) in their EEG data. All participants had normal or corrected-to-normal visual acuity and no reported colorblindness. Participants with corrected vision wore contact lenses instead of glasses to avoid potential artifacts in the pupillometry data. All participants had clinically normal hearing, defined by tone detection thresholds below 20 dB HL at octave frequencies between 250 Hz and 8 kHz, as confirmed by an audiometric screening. Participants gave written informed consent and were compensated for their participation. All study procedures were approved and overseen by the Boston University Charles River Campus Institutional Review Board.

### 2.2 Experimental setup

**Fig. 1.**
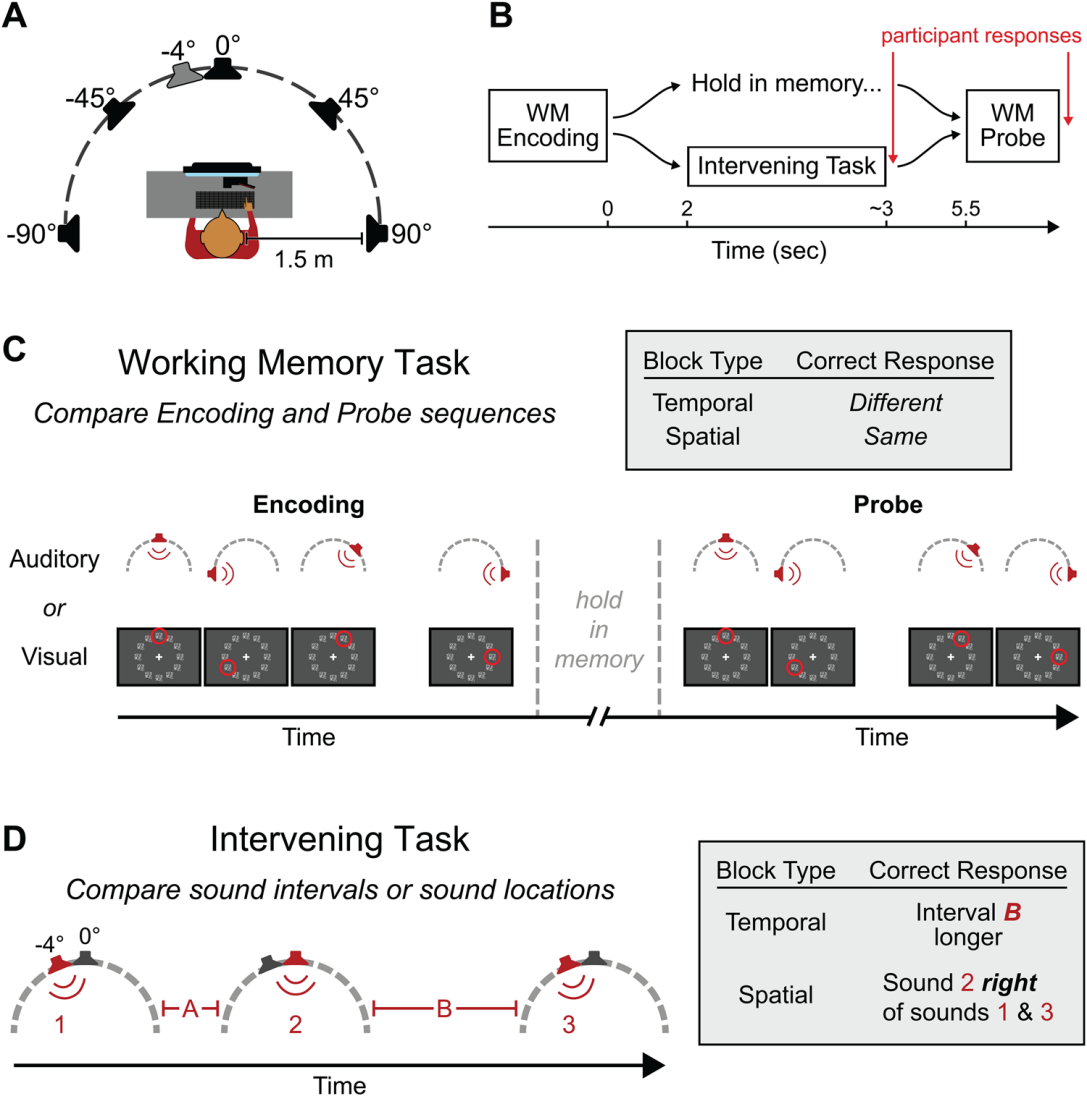
Experimental setup and task design. (A) Depiction of the experimental setup, viewed from above. The loudspeakers at 0°, ±45°, and ±90° azimuth presented stimuli in the WM task, while the loudspeakers at −4° and 0° were used for the Intervening task. (B) Overall dual-task structure. Here and elsewhere, time zero refers to the offset of the final stimulus in the WM encoding phase. (C) WM task structure. Each auditory or visual stimulus is shown in red (sensory modality was held constant throughout a block). Correct responses for this example trial (top right) differ depending on whether the participant was asked to attend temporal or spatial features. Note that in the actual experiment, inter-stimulus intervals were fixed in the auditory-spatial WM task to reduce task difficulty. (D) Auditory Intervening task structure. Participants judged inter-stimulus interval durations in temporal blocks and the relative location of the middle sound in spatial blocks.

The experiment was conducted in a darkened, electrically shielded, sound-treated booth. Participants were seated comfortably with their chin resting on a desk-mounted head support (SR Research). A BenQ 1080p LED monitor (27-inch diagonal, 120 Hz refresh rate) was positioned in front of the participant at approximately 65 cm distance. The monitor was set to 3% of its maximum brightness level to prevent eye fatigue and pupil diameter saturation. An EyeLink 1000 Plus eye-tracking system was placed on the desk just below the display for measurement of pupil diameter. Six free-field loudspeakers (KEF E301) were mounted in an arc around the participant at a distance of 1.5 m. Five of the loudspeakers were equally spaced in azimuth at ±90°, ±45°, and 0° relative to midline; the sixth was placed immediately to the left of the central loudspeaker, at −4° azimuth, and used only in the Intervening tasks described below (Fig. 1A). All six loudspeakers were positioned at approximately 5° elevation relative to the horizontal plane of the eyes to reduce obstruction by the visual display. An RME Fireface UCX soundcard handled auditory stimulus presentation.

A standard keyboard was used to register all task responses. Sixty-four-channel EEG data (Biosemi ActiveTwo system) were collected at a sampling rate of 2048 Hz. Separate PCs were used for pupillometry recording and EEG recording, and a third PC was used for presenting stimuli and registering behavioral responses. To ensure synchrony of event triggers (e.g., trial starts, stimulus presentation) between the pupillometry and EEG data, triggers were output through the S/PDIF channel on the soundcard, converted to TTL pulses using a custom converter box, and written simultaneously into the EEG and pupillometry data files. Experiment control was carried out using custom MATLAB software, and visual stimulus presentation was implemented using the Psychtoolbox package (Brainard, 1997).

### 2.3 Task and experimental design

Participants performed a dual-task paradigm, comprising a working memory (WM) task and an Intervening task (Fig. 1B). Each trial started with a 1.5-second baseline period, followed by the presentation of a sequence of four auditory or visual stimuli to be encoded in WM. Each stimulus was presented at 1 of 5 (auditory) or 12 (visual) locations, and each inter-stimulus interval in the sequence was randomly set to be either short or long (more details below). The WM task could be either temporal or spatial, yielding four total WM task conditions: auditory-temporal (AT), auditory-spatial (AS), visual-temporal (VT), and visual-spatial (VS). When the WM task was temporal, participants were instructed to remember the pattern of inter-stimulus intervals (i.e., the rhythm), regardless of spatial locations. When the WM task domain was spatial, participants had to remember the locations of the stimuli, regardless of order or timing (Fig. 1C). In general, both the locations and intervals could change between the encoding and probe sequences (although an exception was made in the AS WM condition; see below). Participants were instructed to ignore changes in the unattended domain.

Participants retained stimulus information in WM for 5.5 seconds, after which a four-stimulus probe sequence was presented in the same sensory modality as the encoded sequence. Participants compared the encoded and probe sequences and made a same-different judgment on the remembered domain (temporal or spatial). After the conclusion of the probe stimulus, participants had 1.5 seconds to indicate whether the encoded and probe sequences were the same (by pressing “1” on the keyboard) or different (by pressing “0”). Each block contained an equal number of same and different trials, ordered randomly. Participants maintained fixation on a small black cross (0.41° visual angle) at the center of the display throughout the trial. After the trial, a 200 ms visual cue indicated whether the participant’s WM task response was correct (a small circle for correct trials and an “x” for incorrect trials). Between each trial, an eyetracker drift check was performed by measuring eye position while the participant maintained center fixation. If the reported eye position differed from screen center by less than 2°, the experimenter manually advanced to the next trial. If the offset was greater than 2°, the eyetracker was recalibrated. On average, there was approximately 2 seconds between the end of one trial and the start of the next trial’s baseline window.

We conducted pilot testing of the WM tasks to determine parameter settings that avoided ceiling or floor effects in behavioral measures. For participants to perceive the different inter-stimulus intervals equally well, a larger temporal separation was needed for the visual stimuli (200 and 580 ms) than the auditory stimuli (200 and 340 ms). Conversely, the visual-spatial task was too easy with only five stimulus locations, so the number of potential visual locations was increased to 12. Finally, participants struggled to perform the AS WM task when stimulus timing was variable; therefore, both encoding and probe stimuli in this condition were presented isochronously at the longer inter-stimulus interval.

On some trials, participants also performed an Intervening task during the WM retention period. This task was always auditory to allow pupil diameter to be measured in the absence of any visual stimulation, but like the WM tasks, it could be either temporal or spatial (AT or AS; seeFig. 1D). The stimulus structure was the same for the temporal and spatial variants. Starting 2 seconds after the offset of the final stimulus in the WM encoding phase, a sequence of three auditory stimuli was presented. These stimuli were white noise bursts, acoustically distinct from the stimuli used in the auditory WM tasks (tone complexes) to prevent confusion between the tasks. One of the two intervals between the stimuli was randomly chosen to be slightly longer than the other. The precise intervals were jittered on each trial, with an average short interval duration of 370 ms and an average long interval duration of 550 ms (180 ms average difference). The sounds were presented from the two near-frontal loudspeakers (−4° and 0° azimuth). The first stimulus played from one of these loudspeakers, chosen randomly and with equal probability; the second stimulus was played from the other loudspeaker, and the third was played from the same location as the first. In the temporal Intervening task, participants judged whether the first or second inter-stimulus interval was longer. In the spatial Intervening task, participants were asked to determine whether the second sound was to the left or right relative to the first and third sounds. Participants registered Intervening task responses with a keypress immediately after the last Intervening task stimulus. Thus, neural signatures of motor planning and execution may be present in the EEG data near the end of the WM retention phase. However, any motor components in the EEG data should be the same in every condition with an Intervening task, allowing for fair comparison between the different task combinations. No feedback was provided for the Intervening task.

This stimulus design allowed physically identical auditory stimuli to be used for the spatial and temporal Intervening task conditions. However, it did introduce an asymmetry between conditions in the amount of information required to do the task. In the temporal Intervening task, participants needed to attend all three stimuli in order to compare the two inter-stimulus intervals, whereas in the spatial Intervening task, participants were often able to make their judgment on the second auditory stimulus by comparing its location to the first. This could result in a longer period of increasing pupil size in the temporal task, leading to larger peak pupil diameter. In addition, behavioral accuracy differed between the two Intervening tasks. Both of these differences could confound comparisons across the different Intervening tasks; however, our analyses focused mainly on comparisons across the four WM conditions*within*each Intervening task, which minimized the impact that potential differences in difficulty and behavioral strategy could have had on our main conclusions.

Trials were grouped into blocks of 20. Within each block, the WM and (when present) Intervening task conditions were held constant. At the start of each block, an instruction screen indicated the sensory modality and relevant domain (temporal or spatial) for both tasks in the upcoming block. Participants were allowed to take untimed breaks between blocks. Participants performed one block of each WM and Intervening task before any conditions were repeated, and the same condition was not allowed to repeat in adjacent blocks. In total, participants performed 40 trials of each combination of WM task modality, WM task domain, and Intervening task condition.

Each complete dataset required three separate visits to the lab. The first session was reserved for consent, audiometric screening, and task practice. Participants practiced each variant of the WM and Intervening tasks in isolation until they understood the procedure, then performed three to five example trials of the full dual-task paradigm. Data collection for the actual experiment occurred in the two subsequent sessions, with the auditory and visual WM task conditions performed on separate days. The order of these sessions was randomized and counterbalanced across participants.

### 2.4 Stimulus details

For the visual WM tasks, 12 stimuli were arranged in a circle centered on the fixation cross and shown on a constant dark grey background (2.51 cd/m^2^). Each stimulus was a square patch of visual noise, subtending 2.86° of visual angle and composed of a 30 x 30 grid of smaller squares. Each of these smaller squares was filled with a greyscale color between black and white, such that the average luminance across the patch was 5.85 cd/m^2^. The angular spacing between each patch was 30°, and the entire stimulus circle subtended 21.59° of visual angle. To equate display luminance and structure across tasks, these visual stimuli remained present but static throughout the auditory WM and Intervening tasks. In the visual WM conditions, a stimulus event consisted of resampling the luminance of each small square in a given patch; this made the visual patch appear to jitter without changing the average luminance across the patch.

For the auditory WM tasks, each stimulus was a 50-ms tonal chord consisting of 3 harmonically unrelated complex tones (fundamental frequencies of 422, 563, and 670 Hz). Each tone included its first nine harmonics, set to equal amplitude. The same tone complex was used for all auditory stimuli in the WM tasks. For the Intervening tasks, the stimuli were one of five pre-generated, 50-ms bursts of noise bandpass filtered between 100 and 10,000 Hz. Identical noise tokens were used for all three stimuli within each Intervening task sequence. Both types of stimuli were relatively broadband, thus ensuring they provided rich and robust spatial localization cues. All auditory stimuli were ramped on and off with a 5 ms cosine-squared ramp to avoid onset and offset artifacts.

In the visual WM tasks, the first stimulus to change in the encoding and probe sequences was always the one at top-center (12 o’clock). Similarly, in all auditory WM sequences, the first sound was presented from the central loudspeaker. This was done to equate the number of items stored in WM across the spatial and temporal WM tasks; with four stimuli in each sequence, there were three intervals to remember for the temporal tasks, and so the first stimulus location was held constant such that only three locations needed to be remembered for the spatial tasks.

### 2.5 Behavioral data analysis

The primary behavioral metrics in this study were error rates on the Intervening and WM tasks. Behavioral performance was statistically analyzed using logistic mixed-effects regression models. For Intervening task performance, the model included fixed-effect terms for the WM task condition (AT, AS, VT, or VS) and the Intervening task type (AT or AS), as well as the interactions between these terms. Random-effects terms were included to capture participant-specific intercepts and slopes for both predictor variables. The model was structured as follows, with*Int*capturing whether the Intervening task was temporal or spatial.

$$\begin{array}{l} logit(ErrorRate)\sim WMCondition*Int+\\ \;\;\;\;\;\;\;\;\;\;\text{(}1+WMCondition+Int\text{|}Participant) \end{array}$$

A similar model was used to analyze WM recall errors. To facilitate separate examination of effects of WM task modality (auditory or visual) and domain (temporal or spatial), the WM condition was expressed as two fixed-effect terms in this model:

$$\begin{array}{l} logit(Error\;Rate)\sim Modality_{WM}*Domain_{WM}*int+\\ \;\;\;\;\;\;\;\;\;\;\;\;(1+Modality_{WM}+Domain_{WM}+int|Participant \end{array}$$

To investigate main effects and interactions at the group level, the coefficients from these models were fed into a two-way (Intervening task) or three-way (WM task) repeated-measures ANOVA.*p*-Values for model terms were estimated using the Satterthwaite approximation for degrees of freedom. Contrasts were treatment coded with baseline levels initially set as “auditory” for WM modality, “temporal” for WM domain, and “none” for the Intervening task. Pairwise post-hoc testing was conducted by cycling which level was coded as baseline for each factor until a β-weight (and corresponding*p*-value) could be extracted for each necessary pair of conditions.

We did not attempt to precisely equate task difficulty across conditions for individual participants, which makes it difficult to compare performance across Intervening task or WM task conditions. Instead, we assessed how performance on one task (keeping the condition of that task fixed) changed across conditions of the other task. For Intervening task performance, this meant examining performance on each separate Intervening task as a function of the modality and domain of the WM task; for WM task performance, we examined recall in each separate WM condition as a function of the Intervening task condition. For all post-hoc tests, the baseline significance level of α = 0.05 was corrected for multiple comparisons using the Holm-Bonferroni method (Hervé, 2010). Only*p*-values surviving this correction are referred to as “significant” in the Results.

### 2.6 Pupillometry data collection and analysis

Pupil size data were continuously recorded at a sample rate of 500 Hz. A custom MATLAB analysis pipeline was used to prepare pupil size data for statistical analysis. First, trials were split into baseline and trial windows based on triggers in the data. The trial window included the WM encoding and WM retention / Intervening task phases, ending immediately before the WM probe sequence was presented. Next, blinks were automatically detected based on instantaneous position, velocity, and acceleration thresholds. An experimenter manually reviewed the data and, using the GUI, adjusted blink thresholds or manually marked additional blink segments as needed. Blinks and other marked segments of noisy data were replaced with a linear interpolation between the average of the three samples (6 ms) preceding and following the blink. When blinks occurred at the beginning of the trial window, a linear fit was made to the five samples (10 ms) following the blink, and this fit was back-projected through the blink segment. The opposite procedure was used for blinks falling at the end of the trial window. Trials in which more than 25% of the data was made up of rejected segments were automatically excluded from further analysis. When data from both eyes were available, the two traces were averaged. To produce the final output, the traces were concatenated, Z-scored, and then split back into individual trials. This procedure eschewed absolute pupil size measures in favor of values that were individually normalized for each participant, so only relative pupil diameter between conditions is interpreted.

Statistical testing for differences between conditions in the pupil time courses was carried out using non-parametric permutation tests. First, in the data averaged across trials (within conditions and individual participants), the difference between a given pair of conditions was assessed parametrically using a paired T-test at each time point. Next, we performed 2000 iterations of randomly shuffling the condition labels and recomputing the T-tests, generating a null distribution at each time point. Finally, the significance level of the observed difference was determined by calculating the proportion of the null distribution with a T-value equal to or larger than the T-value from the actual data. Significant differences were only considered reliable if the*p*-value fell below 0.05 for a minimum of 15 consecutive samples.

### 2.7 EEG data analysis

EEG data were processed using the FieldTrip package in MATLAB (Oostenveld et al., 2011). For event-related potential (ERP) analyses, EEG preprocessing comprised the following steps: read in the continuous data one channel at a time and immediately downsample to 256 Hz; re-reference the data to the average of two electrodes placed on the mastoids, bandpass filter between 0.5 and 20 Hz (zero-phase FIR filter, transition width of 0.2 Hz, order of 9274) to remove slow drift and any high-frequency noise, respectively; manually identify and remove segments containing muscle artifacts; perform an independent components analysis (ICA) and remove components corresponding to blinks or saccadic eye movements; epoch the data from 100 ms before to 500 ms after each individual auditory or visual stimulus (timing differences between conditions precluded whole-trial averaging); reject any epochs in which the data exceeded a 100 µV peak-to-peak threshold; and baseline correct by subtracting off the mean of the first 100 ms of each epoch. An average of 98.2% of ERP epochs from each recording session survived artifact rejection (minimum 85.6%, maximum 99.9%).

The ERPs of primary interest were those elicited by the Intervening task. Preliminary analysis revealed that effects of WM condition mainly manifested in the P2 component of these ERPs. Therefore, P2 amplitudes were computed for each participant as the average of the ERP waveform between 190 and 220 ms post-stimulus across a cluster of fronto-central electrode sites, where the P2 response was strongest (Fz, FCz, Cz, FC1, and FC2 on the standard 10-20 layout). These data were analyzed using repeated-measures ANOVA with factors of WM task modality, WM task domain, and Intervening task condition.

For time-frequency analyses, a similar preprocessing pipeline was used, but with the following differences. First, the low-pass filter cutoff was raised to 80 Hz. Second, participant average ERPs (recomputed with the new filter cutoffs) were subtracted from the timeseries data at each stimulus timestamp. The goal of this step was to limit the contribution of the evoked response (ERP) to our measurement of the non-phase-locked oscillatory response (Deiber et al., 2009, though trial-to-trial ERP variability may limit the effectiveness of this approach). Subtracted ERPs were specific to each trial phase (i.e., encoding, retention, and probe), WM and Intervening task condition, and position in the stimulus sequence. Third, the data were split into whole-trial epochs, spanning the baseline period through the final stimulus in the probe sequence, instead of shorter individual-stimulus epochs. The continuous Morlet wavelet transform (wavelet width of 5 cycles in 1 Hz steps) was used to obtain the power spectra of each trial. Prior to wavelet analysis, the signal was mirror-padded to avoid edge artifacts during the wavelet transform. This was done by copying the first and last 5 seconds of the epoch, reflecting each copy on the time axis, then appending them to the beginning and end of the signal, respectively. Finally, the data were split into the key trial phases: baseline, WM encoding, WM retention/Intervening task, and WM probe. Trials were removed if the 100 µV peak-to-peak artifact threshold was reached in either the baseline or retention/Intervening task windows. An average of 94.6% of trials from each recording session survived artifact rejection (minimum 84.1%, maximum 100%).

We next extracted time courses from the resulting time-frequency data at channel Pz, where alpha power was strongly modulated during WM encoding and maintenance, at each frequency step within the alpha (8-12 Hz) band. Individual differences in the peak frequency of alpha oscillations are well established (Klimesch et al., 1999). Thus, the individual alpha frequency for each participant was defined as the frequency at which the absolute value of the alpha change relative to baseline was maximal during WM retention (the direction of alpha change was found to flip based on WM modality in the present study). Alpha power time courses were reported as the power at this frequency averaged with the power 1 Hz above and below the individually defined alpha frequency.

Alpha power time courses were analyzed using a non-parametric permutation testing approach, similar to the pupillometry analysis. Paired t-tests were first computed between each pair of conditions at each time point. These t-tests were then recomputed over 2000 iterations of randomly shuffling the condition labels. At each time point, the comparison was considered significant if the actual T-value was larger than 95% of the T-values obtained by random permutation.

## 3 Results

### 3.1 Working memory task performance

**Fig. 2.**
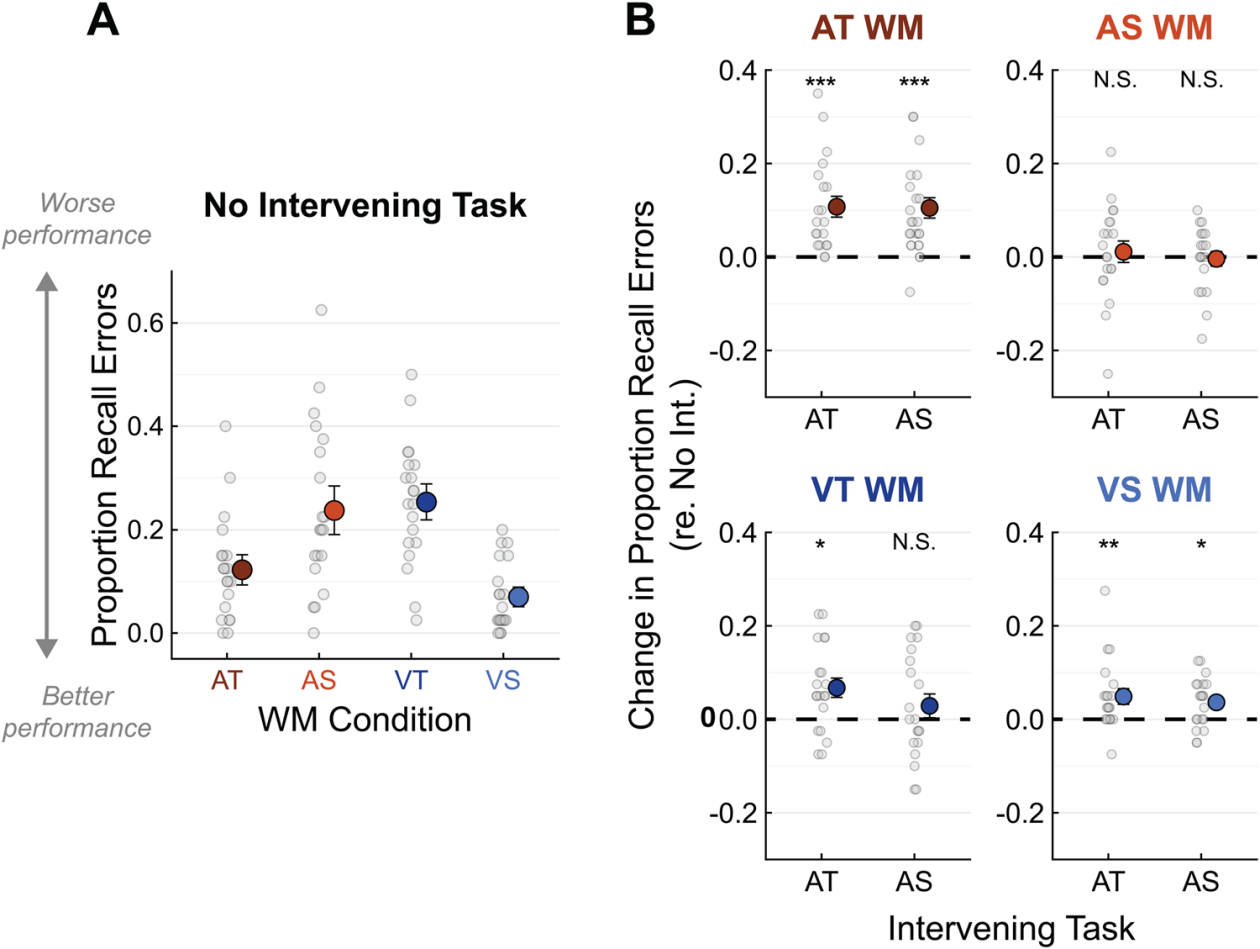
Working memory task performance. (A) The proportion of error trials in each WM task condition with no Intervening task. Chance performance is at 0.5. (B) The change in WM task error rate for each WM and Intervening task combination, relative to the no Intervening task conditions in (A). Asterisks indicate significant differences from the corresponding no Intervening task conditions (no significant differences were found between the two Intervening tasks within any WM condition). * =*p*< 0.05, ** =*p*< 0.01, *** =*p*< 0.001.

The sensory modality and information domain of the WM task modulated the degree of interference caused by the auditory Intervening tasks. These patterns were assessed with a logistic mixed effects model with fixed effect terms of WM task modality, WM task domain, and Intervening task (including the condition with no Intervening task). An ANOVA conducted on the coefficients of this model revealed two significant effects. First, there was a two-way interaction between WM task modality and domain (χ^2^(1,20) = 206.4,*p*< 0.001). This reflects the overall lower error rates in the AT and VS WM task conditions (Fig. 2A), in which the sensory modality was optimally suited for the information domain of the WM task. Second, and more importantly, the ANOVA revealed a three-way interaction between WM modality, WM domain, and Intervening task condition (χ^2^(2,20) = 19.0,*p < *0.001). This interaction indicates that the same auditory Intervening tasks had different effects on WM retrieval depending on the modality and domain of the WM task. These effects were examined via post-hoc testing restricted to comparisons within each WM task condition. These comparisons reflect differences in WM task performance attributable solely to the influence of the Intervening task and not difficulty differences between the WM task conditions.

To visualize memory interference effects,Figure 2Bshows the*difference*between WM task error rates in each condition with an Intervening task and the corresponding condition with no Intervening task (fromFigure 2A). When the WM task was AT (top-left panel), both auditory Intervening tasks significantly impaired WM retrieval (*p*< 0.001 for both). When the WM task was AS, on the other hand, the same Intervening tasks had no detectable impact on WM retrieval (top-right panel). This supported our hypothesis that the AS WM information would be mapped into a representation in the visual-spatial WM network, protecting it from interference from auditory perceptual processing.

However, overall poorer WM recall accuracy in the AS WM condition may have limited our ability to detect interference effects because closer-to-chance performance left less room for recall to be further impaired by the Intervening tasks. To explore this possibility, we tested whether participants who made fewer AS WM task errors in the absence of an Intervening task (Fig. 2A, orange)—leaving more room for interference effects—would show greater interference from the Intervening tasks (Fig. 2B, top-right). This correlation approached significance, but it was driven by one outlier participant who performed worse than chance on the AS WM task overall. With this outlier participant removed, there was no significant relationship (R^2^= 0.003,*p*= 0.76, combined across the AT and AS Intervening tasks; not shown). Thus, the finding that the auditory Intervening tasks had minimal impact on participants’ ability to recall AS information from WM did not seem to be driven by ceiling effects.

**Fig. 3.**
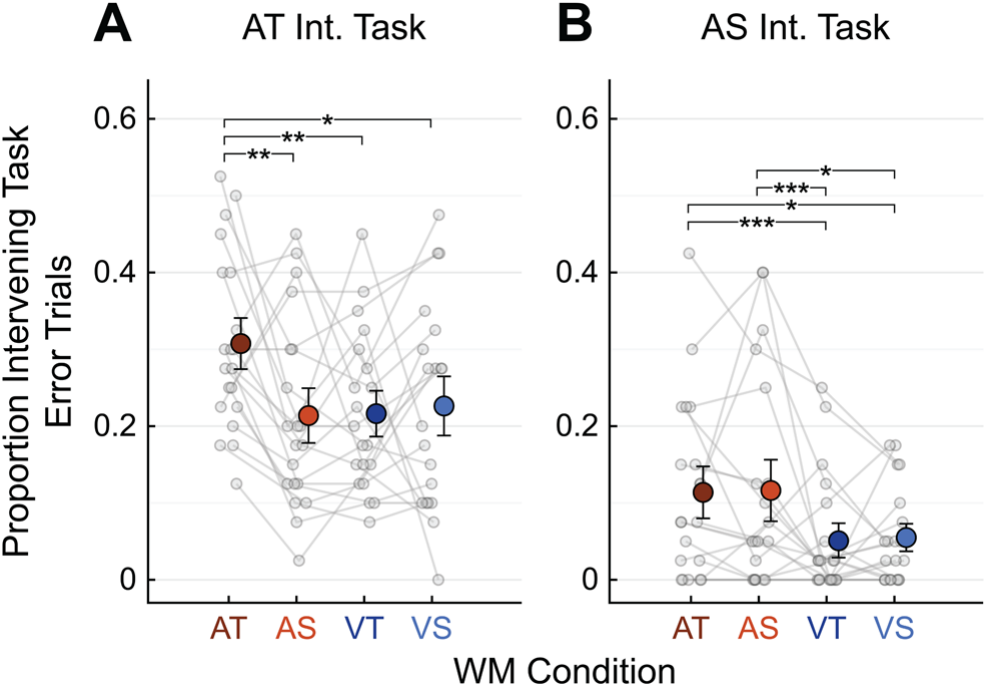
Intervening task error rates. Performance is shown for the AT (A) and AS (B) Intervening tasks. Grey points represent individual participants, large colored circles represent means, and error bars represent S.E.M. Chance performance is an error proportion of 0.5. Asterisks indicate significant differences between WM task levels in the mixed-effects model, examined separately within each Intervening task. * =*p*< 0.05, ** =*p*< 0.01, *** =*p*< 0.001.

Retrieval of visual information from WM was also modestly impaired after performing the auditory Intervening tasks (Fig. 3B, bottom panels). These effects were weaker than the interference caused by dual-task load on the auditory-temporal network in the AT WM condition. In all WM conditions, patterns of interference did not differ significantly between the AT and AS Intervening tasks.

### 3.2 Intervening task performance

Holding information in WM also interfered with auditory perceptual processing in the Intervening task, to a degree mediated by the sensory modality and information domain of the WM task. Intervening task performance was analyzed using a logistic mixed-effects model with fixed-effect terms of Intervening task condition (AT or AS) and WM task condition (AT, AS, VT, or VS). An ANOVA conducted on the model coefficients showed significant main effects of Intervening task condition (χ^2^(1,20) = 32.7,*p*< 0.001) and WM task condition (χ^2^(3,20) = 17.6,*p*< 0.001), as well as a significant interaction between these factors (χ^2^(3,20) = 24.1,*p*< 0.001). The main effect of Intervening task reflects overall lower error rates (better performance) on the AS compared to the AT Intervening task. This is a consequence of the exact stimuli used; specifically, the two loudspeakers used for the Intervening tasks were placed as close together as possible, but the spatial separation (4°) was nonetheless large enough for the AS Intervening task to be relatively easy for some participants. More importantly, the interaction between WM task condition and Intervening task condition indicates that the type of information held in WM affected perceptual processing differently depending on whether the Intervening task was temporal or spatial. These patterns of perceptual interference were further analyzed with post-hoc testing restricted to comparisons within each Intervening task.

Participants made significantly more perceptual errors on the AT Intervening task when the information being held in WM was also AT, as compared to all the other WM conditions (Fig. 3A; AT vs. AS,*p*< 0.001; AT vs. VT,*p*< 0.001; AT vs. VS,*p*= 0.005). The pattern of interference was similar for the AS Intervening task condition, except that the error rate was also elevated when the information held in WM was AS, matching the WM task (Fig. 3B). Overall, for the AS Intervening task, interference tended to be higher when both task were auditory than when the information in held in WM was visual (AT vs. VT,*p *< 0.001; AT vs. VS,*p=*0.004; AS vs. VT,*p *< 0.001; AS vs. VS,*p=*0.003).

Importantly, these patterns of Intervening task performance cannot be explained by differences in task difficulty between the WM conditions. Errors on both auditory Intervening tasks were relatively high when the auditory-temporal WM network was loaded (AT WM condition), but participants made relatively few recall errors in this WM task condition when there was no Intervening task (Fig. 2A). Conversely, auditory Intervening task performance was impacted relatively little by holding VT information in WM, a condition in which WM recall errors were relatively high. Thus, rather than being determined by the combined difficulty of the two tasks, perceptual interference in Intervening task performance was driven by patterns of modality- and domain-based interference with the information participants were holding in WM.

### 3.3 Pupil dilation

**Fig. 4.**
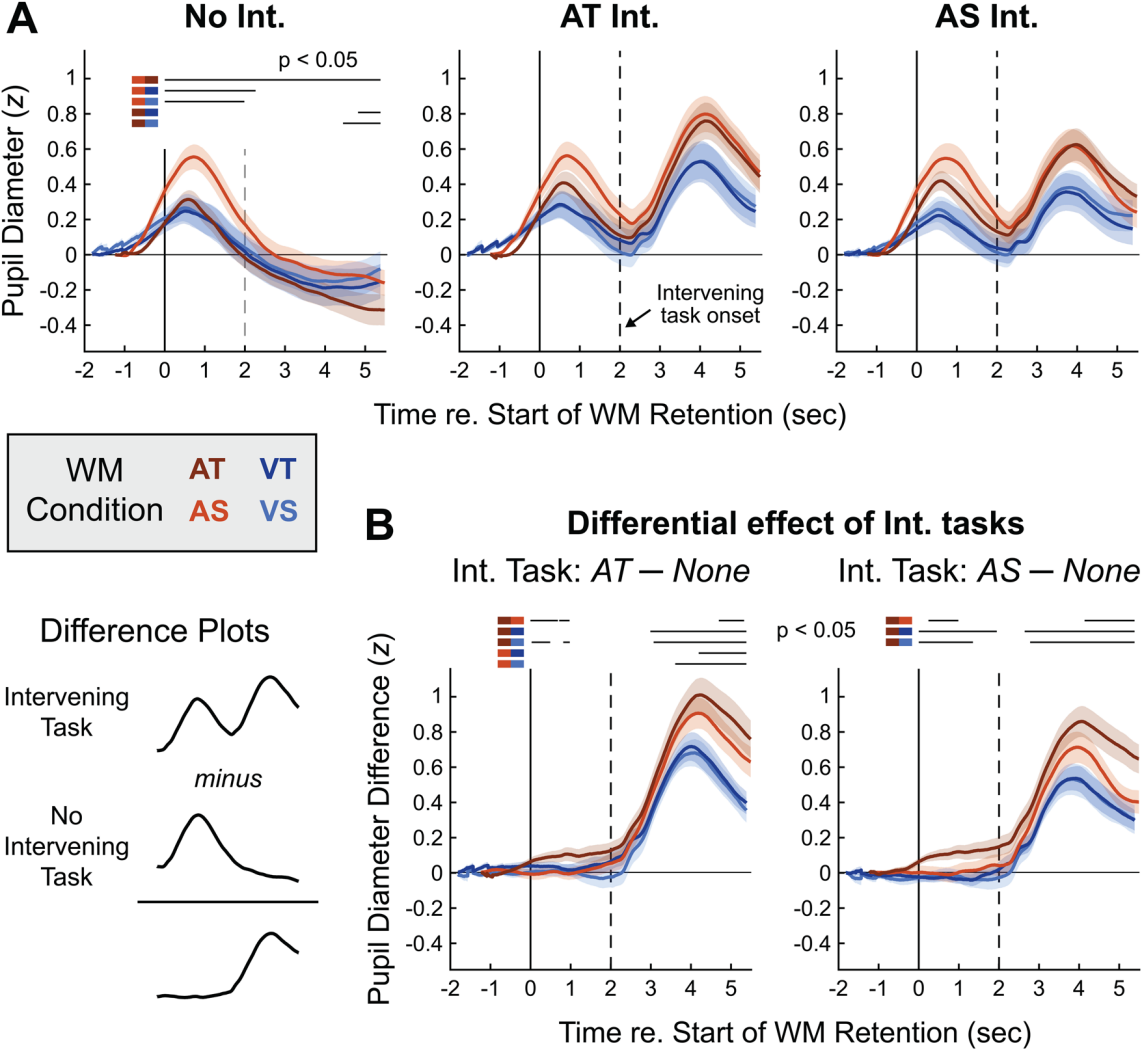
Pupillometry time courses. (A) Grand average Z-scored pupil responses are shown in the No Intervening task condition (left) and elicited by physically identical stimuli in the AT (middle) and AS (right) Intervening tasks. Solid vertical lines represent the end of the WM encoding sequence, while dashed vertical lines indicate the onset of the Intervening task (when present). Error clouds represent S.E.M. Horizontal black lines above the traces represent time regions of significant difference in permutation testing, with the two WM conditions being compared indicated by colors next to each significance line. (B) The difference between pupil responses elicited in conditions with an Intervening task and the corresponding WM conditions with no Intervening task.

Pupil dilations indexed task effort while participants encoded stimulus information in WM and performed the auditory Intervening tasks. Prior to Intervening task onset, pupil dilations corresponding to WM encoding peaked roughly 700 ms after the end of the encoding sequence, consistent with the timing from other pupillometry studies (e.g.,Winn et al., 2018). Interestingly, encoding AS information in WM elicited significantly larger pupil dilations than any of the other WM conditions (Fig. 4A, left panel). Of note, a comparable pupil size increase was not observed in the VT WM condition, despite similarly high WM recall errors in this condition. This indicates that encoding auditory-spatial information in WM—which likely recruited the visual-spatial WM network—may be particularly effortful.

Performing either auditory Intervening task elicited a second pupil dilation, the amplitude of which scaled with the modality and domain of the information participants were holding in WM (Fig. 4A, middle and right panels). To better isolate the impact of the Intervening tasks on pupil diameter, we subtracted individual participant pupil responses in the no-Intervening task condition from their corresponding Intervening task responses (Fig. 4B). Statistical differences between these differential responses were assessed using non-parametric permutation testing, restricted to a time window spanning the WM retention phase (time zero and later inFig. 4). We first tested whether task effort would reflect the overall difficulty difference between the AT and AS Intervening tasks by averaging pupil traces across the four WM conditions and comparing Intervening task responses (not shown). This confirmed that pupil dilations elicited by the AT Intervening task were larger than those elicited by the AS Intervening task (*p*< 0.05 for all time points after 3.7 seconds), matching the higher behavioral error rates on the AT Intervening task.

In our main analysis, we compared the effect of the type of information being held in WM on the second pupil dilation during each of the Intervening tasks (i.e., for each Intervening task we compared the differential pupil traces for the four WM conditions;Fig. 4B). The pattern of these difference traces was similar between the AT and AS Intervening tasks. For both Intervening tasks, pupil dilations were larger when the WM task was AT (taxing auditory-biased cognitive resources) and smaller when the WM task was visual (such that the two tasks were presented in different sensory modalities). When the WM information was AT, differential pupil size was also elevated prior to the onset of both Intervening task conditions, indicating a preparatory effort increase in anticipation of potential WM interference. When the WM task was AS, pupil size was intermediate between the AT and the visual WM conditions, suggesting a partial alleviation of Intervening task effort when the WM information could be distributed across auditory-temporal and visual-spatial networks. In the visual WM conditions, pupil dilations were insensitive to whether the WM task was temporal or spatial, similar to the pattern of Intervening task behavioral errors.

### 3.4 Event-related potential (ERP) amplitudes

Event-related potentials (ERPs) elicited by each stimulus event in the WM and Intervening tasks are shown inFigure 5A. ERPs in the encoding and probe sequences of the WM task were not systematically modulated by the Intervening task conditions. However, during the Intervening task—when participants were simultaneously performing the Intervening task and holding information in WM—ERP amplitudes varied depending on the type of information participants were holding in WM. This effect of WM condition was clearest for ERPs elicited by the first sound in the Intervening task sequence, highlighted inFigure 5A. Note that the these auditory Intervening task ERPs differed in overall morphology from ERPs elicited by the auditory WM task stimuli; Intervening task responses had a relatively small N1 component and a relatively large P2 component, possibly due to stimulus differences between the tasks. This issue is revisited in the Discussion.

**Fig. 5.**
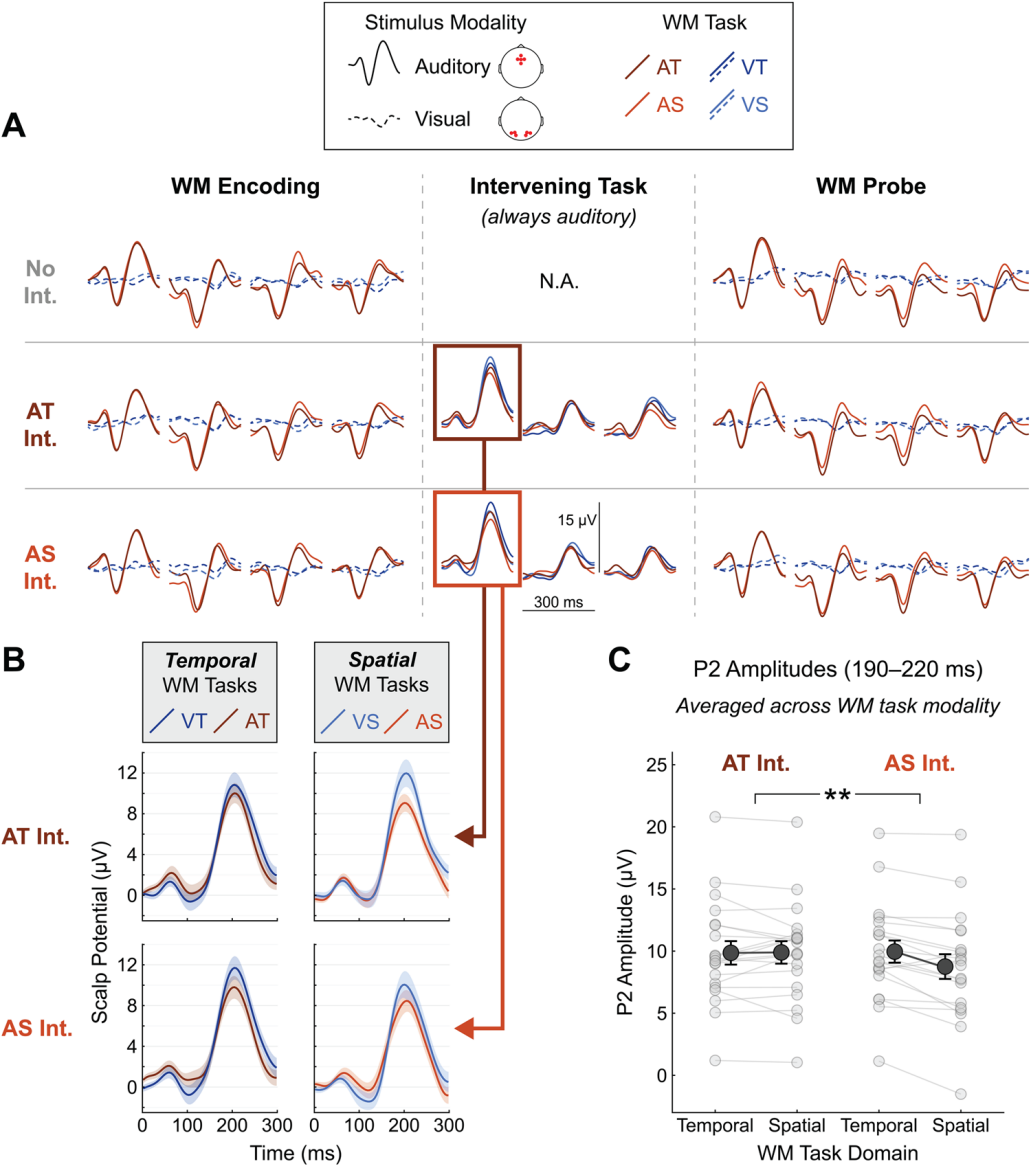
Event-related potentials. (A) Grand-average ERPs for each combination of WM task (colors), Intervening task (rows), and position in the stimulus sequence. Stimulus modality is indicated by solid versus dashed lines. Axes are shown in the top row for scale. Electrodes averaged to produce these ERPs differed by stimulus modality: on the standard 10–20 layout, these were channels Fz, AFz, Cz, F1, and F2 for auditory stimuli and O1, O2, PO3, PO4, PO7, and PO8 for visual stimuli. (B) Intervening task onset ERPs for each combination of WM and Intervening task conditions. (C) ERP amplitudes for each participant (light grey) and averaged across participants (dark grey), collapsed across WM task modality. Error clouds and bars represent S.E.M. ** =*p*< 0.01.

Both the modality and domain of the information stored in WM affected the amplitude of the P2 component of ERPs elicited by Intervening task onset (Fig. 5B). P2 amplitudes were analyzed using a three-way ANOVA with explanatory factors of WM modality, WM domain, and Intervening task domain. This analysis revealed a significant main effect of WM modality (F(1,19) = 7.27,*p=*0.014, η^2^= 0.038) and a significant interaction between WM domain and Intervening task domain (F(1,19) = 10.83,*p=*0.004, η^2^= 0.005). The main effect of WM modality reflects the fact that P2 amplitudes elicited by the auditory Intervening tasks were smaller when the information stored in WM was also auditory than when it was visual (Fig. 5B). The interaction signals an additional effect of similarity in information domain between the two tasks, which was evident primarily when the Intervening task was spatial (AS). The amplitudes of ERPs elicited by the AS Intervening task were consistently smaller when the WM task was also spatial as compared to when it was temporal, shown collapsed across sensory modality inFigure 5C.

Although a three-way interaction involving WM task modality did not reach statistical significance, it appeared that the domain similarity effect was driven mainly by the visual WM conditions. On average, P2s elicited by the temporal Intervening task (AT) were largest in the visual-*spatial*WM condition, whereas P2s elicited by the spatial Intervening task (AS) were largest in the visual-*temporal*WM condition. In support of this, the interaction between task domains was still present in a separate follow-up ANOVA restricted to the visual WM conditions (F(1,19) = 10.77, η^2^= 0.009,*p=*0.004; beneath a Holm-Bonferroni-corrected alpha criterion of 0.017 to account for two additional ANVOAs restricted to the auditory and visual WM conditions). This interaction did not reach significance in a corresponding ANOVA with only the auditory WM conditions, in which ERPs were more suppressed in general. Together, these ERP results suggest a weaker neural response to the Intervening task stimuli when WM was pre-loaded with information that overlapped in sensory modality or information domain with the Intervening task demands.

### 3.5 Alpha-band oscillatory activity

We next examined whether an alpha power signature of WM maintenance would be disrupted by performing an Intervening task during WM retention, and whether this effect would be mediated by modality and domain similarity between the tasks. Unexpectedly, preliminary analysis of alpha power using cluster-based permutation testing yielded no significant effects involving the information domain of the WM task, so data were collapsed across this factor in the subsequent analyses.

**Fig. 6.**
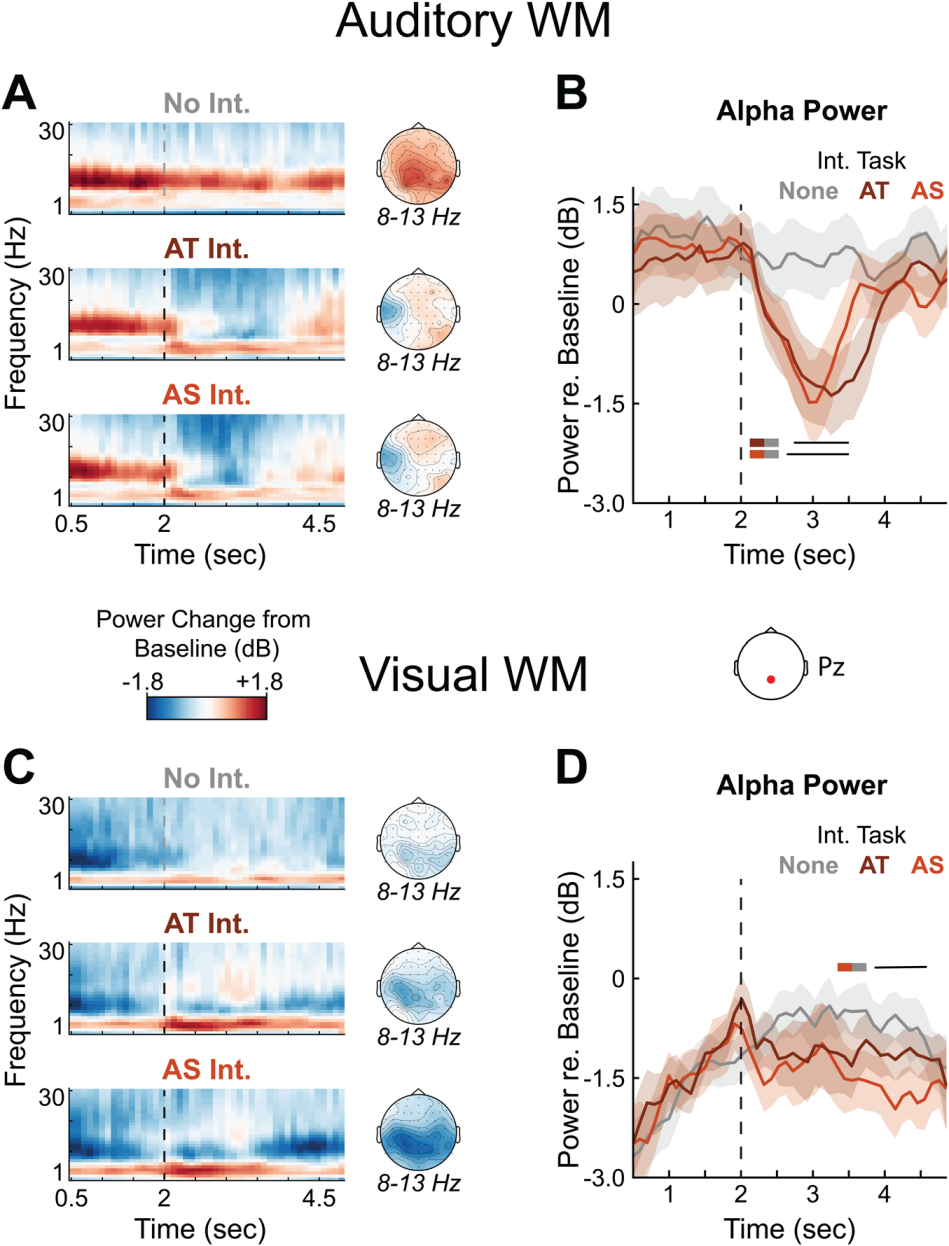
Alpha power during WM retention. Grand-average time-frequency responses are shown at a parietal electrode site (Pz) during the memory retention window in the auditory (A) and visual (C) WM conditions. Responses are averaged across WM domain and shown as dB change relative to the average pre-trial baseline period across conditions. Vertical dashed lines represent Intervening task onset when present. The first and last 500 ms of the retention window were excluded to limit power contributions from responses evoked by the WM task stimuli. Scalp topographies of alpha power throughout the retention window are shown to the right. Grand average alpha power time courses are also shown for the auditory (B) and visual (D) WM conditions. Power time courses for each participant were calculated at their peak alpha frequency ±1 Hz. Error clouds represent S.E.M. Black horizontal bars indicate significant time regions (*p*< 0.05) in permutation testing for the comparisons indicated by colored boxes to the left.

In the auditory WM conditions, alpha power increased relative to baseline during WM encoding (not shown) and remained elevated throughout WM retention when there was no Intervening task, as expected (Fig. 6A). The onset of either of the auditory Intervening tasks suppressed these ongoing alpha oscillations. We examined this effect by conducting cluster-based permutation tests on the alpha power time courses in two time regions of interest: one spanning the Intervening task itself (2 to 3.5 seconds into WM retention, including the Intervening task behavioral response), and another immediately after the Intervening task (3.5 to 5 seconds) when participants needed to start retrieving the information stored in WM to compare to the WM task probe sequence. During both of the Intervening tasks (the earlier window), alpha power was significantly reduced relative to the no-Intervening task condition (horizontal bars inFigs. 6Band6D). There were no significant differences between alpha time courses in the AT and AS Intervening task conditions. In other words, the need to perform the Intervening task caused a disruption of the alpha signature of WM retention/rehearsal, and this disruption was similar between the two auditory Intervening tasks. No significant clusters were found in the later time window (3.5–5 seconds).

In the visual WM conditions, alpha power was suppressed relative to baseline during WM encoding (not shown). From this suppressed point, alpha power increased during WM retention, similar to the auditory WM conditions (Fig. 6C, top panel). On trials with an Intervening task, the suppression of alpha power appeared to resume after the conclusion of the Intervening task, perhaps reflecting an anticipatory shift of top-down attention to prepare for the upcoming visual WM probe stimuli. We examined the alpha power time courses using permutation testing restricted to the same two time windows analyzed in the auditory WM conditions. Unlike for auditory WM tasks, no significant differences were observed during Intervening task performance when visual information was held in WM. However, in between the Intervening task and the WM probe sequence (3.5–5 seconds), alpha power was significantly reduced when the Intervening task was AS—but not when it was AT—relative to when there was no Intervening task (Fig. 6D). This could reflect taxation of visual-spatial cognitive resources when both tasks were spatial, similar to the effects on ERP amplitudes. However, in a direct comparison, differences in alpha power between the AT and AS Intervening task conditions did not reach significance, so this result should be interpreted with caution.

## 4 Discussion

In this study, we investigated interference between perceptual processing and WM maintenance while varying the similarity between tasks in sensory modality and information domain (spatial vs. temporal processing). Convergent evidence from behavior and pupillometry, the latter indexing cognitive effort, showed that the highest degree of interference occurred when the two tasks drew upon shared cognitive resources. Specifically, when both tasks were auditory-temporal (AT) and therefore relied on overlapping attention and WM resources, error rates on the Intervening task were highest, WM retrieval was poorest, and pupil dilations were largest. When the WM task was auditory-spatial (AS), recall was no longer disrupted by interference from the auditory Intervening tasks. This condition will receive special attention below, as we expected that encoding and maintaining AS information in WM would be distributed between auditory- and visual-biased attention and WM networks. When the auditory Intervening tasks were paired with a visual (VT or VS) WM condition, behavioral interference was relatively low and pupil size was smallest. However, in these visual WM conditions with reduced competition for shared resources, subtler patterns of interference based on similarity in task domain (temporal vs. spatial) were nonetheless observed in ERP amplitudes and alpha-band oscillatory dynamics related to WM maintenance.

### 4.1 Behavioral interference patterns support modality and information domain specializations of lateral frontal control networks

The design of this experiment was inspired by the functional configuration of attention and WM networks in the human lateral frontal cortex (LFC). Subregions of the LFC preferentially contribute to auditory or visual processing, but are also recruited by stimuli in the non-preferred modality depending on whether the task is temporal or spatial. However, previous fMRI research has shown that such across-network recruitment is asymmetrical; auditory-spatial processing recruits the visual-biased control network to a greater extent than visual-temporal processing recruits the auditory-biased network (Noyce et al., 2017,^2022^). This sets up a special significance of the AS WM condition in the current study: we expected that WM processing in this condition would rely on both control networks, and that auditory information would (at least partially) be mapped to, and maintained in, the visual-biased network. This predicts that the auditory Intervening tasks would cause reduced interference when AS (as compared to AT) information is held in WM, as the distributed representation of the AS information would reduce demands on the auditory-biased network. This prediction was borne out in behavioral interference in both tasks: Recall was impaired by the auditory Intervening tasks in the AT (but not the AS) WM condition, and errors on the AT Intervening task were elevated with AT (relative to AS) information held in WM.

If the visual-*temporal*(VT) WM condition strongly recruited the auditory-biased network (mirroring AS recruitment of the visual-biased network), one would have expected that VT information would suffer more interference from the auditory Intervening tasks than VS information. However, little behavioral interference was found in either of the visual WM conditions. This result is also consistent with the asymmetric recruitment pattern of the LFC control networks. Since VT processing only weakly recruits the auditory-biased network, we propose that both of the visual WM conditions relied mainly on the visual-biased network, allowing this information to be segregated and protected from interference from the auditory Intervening tasks. Taken together, these results support the joint specialization of WM networks for both sensory modality and information domain (as indicated by previous fMRI studies) and demonstrate the behavioral relevance of this network organization.

Interference effects based on information domain were not symmetrical between the Intervening and WM tasks. Specifically, the domain of the WM task in the auditory conditions (AT vs. AS) affected both WM recall and Intervening task performance, whereas interference effects were largely similar (at least behaviorally) between the AT and AS Intervening tasks. This likely points to important differences in the spatial processing demands of the AS WM task and the AS Intervening task. To perform the AS WM task, participants needed to remember absolute auditory stimulus locations mapped onto physical space. This may have placed more demand on the visual-biased LFC network than did the AS Intervening task, which required only an immediate, relative judgment about auditory spatial positions within each trial, and did not require the participant to map the stimuli to perceived exocentric locations nor remember their spatial positions. While the AS Intervening task certainly involved a form of spatial processing that could interfere with other spatial tasks, it seems likely that it primarily taxed sensory processing taking place within the auditory-biased LFC network, similar to the AT Intervening task. This would explain why both Intervening tasks strongly interfered with recall of AT information from WM, and why pupil dilations elicited by both Intervening tasks showed the highest effort in the AT WM condition. This pattern of results is a consequence of the specific tasks used in this study; if the AS Intervening tasks had a stronger spatial memory component or required participants to map auditory stimuli to external locations, interference patterns may have diverged based on Intervening task domain, similar to what was found between the AT and AS WM task conditions.

Our dual-task approach was designed such that the Intervening task would either increase the load on the active WM network (e.g., when both tasks were auditory-temporal) or require the participant to switch networks between tasks. One lens through which to view the behavioral results is that the processing costs of loading onto a single WM network were greater than the costs of switching between networks. In addition to dual-task interference, task-switching also incurs a processing cost (Arnell & Jolicœur, 1999;Chun & Potter, 2001;Hsieh & Allport, 1994;Meiran et al., 2000), which may have been exaggerated when the two tasks relied on different networks. Our auditory Intervening tasks modestly impaired the recall of visual information from WM, consistent with some behavioral cost of switching between WM control networks. However, this effect was relatively weak compared to the behavioral (and physiological) costs when the tasks loaded onto shared network resources.

While we are confident that our WM tasks engaged sensory-biased networks in the LFC, this alone does not rule out other neural substrates for the interference effects we observed. In particular, some studies have shown that information stored in WM is represented in early sensory brain areas, in both the auditory (Brechmann et al., 2007;Huang et al., 2016;Linke & Cusack, 2015) and visual (Ester et al., 2009;Harrison & Tong, 2009) modalities. Several studies have shown that disrupting activity in the primary visual cortex using transcranial magnetic stimulation during WM maintenance interferes with participants’ ability to recall visual information (see meta-analysis inPhylactou et al., 2022). Additionally, WM load can interfere with perceptual processing (and vice versa) in early sensory cortex. For instance, loading visual short-term memory reduces responses to contrast stimuli in early visual cortex (Konstantinou et al., 2012). Conversely,Deutsch et al. (2023)found that information held in auditory WM could be decoded from activity in the auditory cortex, but not when additional auditory stimuli were presented during WM maintenance. These studies point to early sensory brain areas as another potential locus of interference between WM and perceptual processing. Since activity in the sensory-biased LFC regions is functionally linked to activity in posterior sensory and attention regions (Michalka et al., 2015), an important topic for future imaging studies is to determine where along the processing hierarchy these resource bottlenecks arise.

### 4.2 Pupillometry shows that similarity in sensory modality and information domain increases dual-task effort

Our pupillometry data provided insights about the magnitude and time course of effort as participants maintained stimulus information in WM and performed the different Intervening tasks. When participants performed the WM task alone (with no Intervening task), pupil dilations during WM encoding were largest in the AS WM condition. On one hand, recall errors were relatively common in this condition, and so larger pupil dilations may have been linked to task difficulty. However, a similarly high behavioral error rate in the VT WM condition was not reflected in the pupil data, which calls such an explanation into question. The AS WM condition was distinct in this study in that information could be represented in both the auditory- and visual-biased WM networks. Therefore, an alternative explanation is that leveraging inherently visual resources to represent auditory-spatial information may be especially effortful, even if the exertion of this effort leads to robust encoding of AS information. There is some precedent for this in other types of processing, in which conditions that require the investment of extra effort can also yield perceptual benefits. For instance, using linguistic context to fill in masked words improves speech comprehension but requires effort (Winn & Moore, 2018), and integrating visual information in speech processing improves intelligibility (Sumby & Pollack, 1954) but may be effortful under certain conditions (Strand et al., 2020). The apparent effort required to simultaneously recruit multiple WM networks warrants further investigation, ideally in a paradigm in which task difficulty is more tightly controlled across WM conditions.

The main pupil responses of interest in this study were those elicited by the auditory Intervening tasks under different types of WM load. When analyzed relative to pupil responses in the no Intervening task condition (Fig. 4B), the pattern of pupil responses was broadly consistent with our predictions (informed by previous fMRI studies) and the behavioral data. Each auditory Intervening task elicited the largest pupil response when WM was loaded with AT information, which maximally taxed auditory-biased WM resources needed to process the information in the Intervening task. Conversely, the same auditory Intervening tasks elicited smaller pupil dilations with visual information held in WM, in which case the two tasks relied predominantly on separate neural networks. Relative pupil size was intermediate under an AS WM load, likely again representing the effort of maintaining a representation across multiple WM networks, even though this distributed representation ultimately reduced the influence of auditory interference on recall of AS information.

In previous studies, pupil responses have provided useful insights into the temporal dynamics of effort allocation in cognitive processes that unfold through time, such as decision making (Satterthwaite et al., 2007) and coping with talker variability (Lim et al., 2021). In the speech perception literature, difficult listening conditions—such as when semantic context is removed (Winn, 2016) or when a talker speaks at a fast rate (Winn & Teece, 2021)—produce pupil dilation that lingers well after the stimulus is over. In this study, a similar lingering elevation of pupil size (lasting to the end of the WM retention window) was observed after the difficult AT WM condition. This suggests a reduced ability to recover from interference in this condition, although it should be noted that the slope of pupil size returning toward baseline was similar across WM conditions. We also observed an*anticipatory*increase in pupil size when the WM task was AT, prior to the onset of the Intervening task. Participants likely exerted increased effort rehearsing or protecting WM information when they expected it to suffer interference from an auditory Intervening task (recall that the conditions were blocked, so participants knew to expect this in advance).

Pupil dynamics in this study likely encapsulated both patterns of dual-task interference and the difficulty of the individual tasks. Many studies have shown that pupil size scales with task difficulty (Bijleveld et al., 2009;Kahneman & Beatty, 1966;Porter et al., 2007;van der Meer et al., 2010;Zhao et al., 2019), at least up to the point that the task becomes so difficult that participants disengage from it (Granholm et al., 1996;Ohlenforst et al., 2017). In line with this, overall pupil size (across WM conditions) was larger in response to the more difficult AT Intervening task than the AS Intervening task. However, most of the patterns of pupil size we observed cannot easily be explained by task difficulty. For instance, Intervening task pupil dilations were largest while participants held AT information in WM, but baseline WM recall was more accurate in the AT than the AS or VT WM conditions. A more parsimonious explanation, consistent with the behavioral data, is that these pupil responses were linked to similarity in sensory modality and (when the WM task was auditory) information domain between tasks.

### 4.3 Neural measures reveal additional domain-based interference patterns

Previous dual-task studies showed that concurrent tasks presented in the same sensory modality interfere more with one another than tasks presented in different modalities (Morrison et al., 2015;Scerra & Brill, 2012). A key insight from the current work is that a shared information domain across tasks also increases interference, and that the interaction between modality- and domain-based interference follows patterns predicted by WM networks identified in human fMRI experiments. In the behavioral and pupillometry data, domain-based interference manifested mainly as differences between the AT and AS WM conditions. Interference was generally low in the visual WM conditions (regardless of information domain) because the Intervening tasks were always auditory, and behavioral interference was similar between the AT and AS Intervening tasks due to the limited spatial processing requirements of the AS Intervening task. Nonetheless, in the visual WM conditions, ERPs elicited by the Intervening task stimuli were sensitive to subtle patterns of interference based on the interaction between the domain of WM task (VT vs. VS) and the domain of the Intervening task (AT vs. AS), even though these effects did not rise to the level of affecting behavioral performance or task effort. Specifically, the P2 components of ERPs elicited by Intervening task stimuli were smaller when the information domain of the Intervening task matched that of the WM task.

We interpret the variations in P2 component amplitudes as a reduction in neural response strength when the two tasks competed for shared WM and attentional resources. Previous studies have found similar reductions in ERP amplitudes when stimuli are processed under WM load. For instance, the amplitudes of ERPs elicited by to-be-remembered visual stimuli decrease systematically as more stimuli are added to WM (Agam & Sekuler, 2007). In a dual-task setting, remembering auditory information has been shown to reduce the amplitude of early visual responses, including the P1, N1, and P2 components of ERPs elicited by a primary visual task (Gherri & Eimer, 2011;Pratt et al., 2011). A common interpretation of such results is that the domain-general attention required to encode and maintain information in WM reduces the participant’s capacity to attend additional sensory inputs, resulting in weaker sensory responses. In the current study, rather than manipulating the presence of a WM task or the number of items to be remembered, we manipulated whether two tasks relied on shared or separate WM and attention control networks. Our ERP results suggest that loading WM and perceptual tasks onto the same control network (via the tasks sharing a common sensory modality and/or information domain) creates competition for neural resources that can weaken neural responses to task-relevant sensory inputs.

The auditory Intervening tasks also elicited smaller P2 components when auditory as compared to visual information was held in WM. This reflects the pattern of modality-based interference found in the behavioral data, with increased neural interference when the two tasks competed for auditory-biased resources. However, an alternative account for this result could be that stimuli presented during auditory WM encoding caused adaptation of neural responses, resulting in suppressed responses to the onset of the auditory Intervening task. We do not suspect this for two reasons. First, response adaptation is not evident across the auditory ERPs in the WM encoding sequence (seeFig. 5A). Second, although the 2-second gap between the end of WM encoding and Intervening task onset was close enough that adaptation could theoretically have had some effect on the ERPs (Budd et al., 1998), neural responses would have likely been reset by the acoustic differences between the WM and Intervening task stimuli, as is observed in auditory oddball paradigms (Paavilainen, 2013). Regardless, neural adaptation cannot easily account for differences in response strength based on information domain, as the temporal and spatial task variants used physically identical stimuli.

The Intervening task ERPs had a different overall morphology than ERPs elicited by the auditory WM task stimuli (seeFig. 5A), with smaller N1 components and more pronounced P2 components. These differences in auditory ERP morphology between the WM and Intervening tasks may have resulted from acoustic differences between the stimuli (tone complexes in the WM task, white noise bursts in the Intervening task). At least one study has shown a larger positive-going component in ERPs elicited by white noise bursts compared to pure tones (Matsuo et al., 2019), and parallels can be drawn between the Intervening task ERPs in this study and the cortical responses to noise-like fricative phonemes inKhalighinejad et al. (2017).

Beyond the P2, the Intervening task ERPs also showed a small positive component that peaked around 350 ms post-stimulus (seeFig. 5B). This appears to be P3 response, which is typically elicited by either detection of a target stimulus amid distractors (P3b), or the presence of a distinct, unexpected stimulus (P3a;Escera & Corral, 2007;Escera et al., 1998;Friedman et al., 2001;Squires et al., 1975). Since the Intervening task stimuli were task-relevant and either acoustically distinct from the WM task stimuli (auditory WM conditions), or the first auditory stimulus encountered on the trial (visual WM conditions), a P3 response might be expected in these ERPs. A substantial body of work has demonstrated that P3 amplitudes are modulated by dual-task demands. A central finding in this literature is that as the cognitive demand of a primary task increases, the P3 component of stimuli elicited by a secondary task decreases; in other words, there appears to be an attentional tradeoff between tasks that require shared resources (Nash & Fernandez, 1996;Natani & Gomer, 1981;Sirevaag et al., 1989). Compatible results have been shown using N-back tasks, which require participants to attend a sequence of stimuli, comparing each to previous stimuli stored in WM. As the memory load is increased (by increasing the number of positions back, “N”, that need to be remembered), P3 amplitude decreases (Watter et al., 2001). Qualitatively, P3 amplitudes in our study appear to be in accord with these previous reports, in that P3 amplitudes tended to be smaller when both tasks were auditory as compared to when the WM task was visual (seeFig. 5B), perhaps due to increased competition for attention and WM network resources. However, our task was not prospectively designed to elicit the P3, and difficulty differences between the WM task conditions render interpretation of this component speculative. Future dual-task studies could employ paradigms known to reliably elicit the P3, such as oddball detection paradigms, to leverage the strong theoretical grounding of how this component behaves under dual-task load to test hypotheses about interference patterns.

Finally, we observed modulations in oscillatory alpha power based on the modality of the WM task and the information domain of the Intervening task. The largest alpha power changes from baseline were found at central-parietal electrode sites. Alpha activity with this scalp distribution has been linked to maintaining various types of information in WM, including visually presented letters and numbers (Jensen et al., 2002;Klimesch et al., 1999;Schack & Klimesch, 2002), shapes (Herrmann et al., 2004;Johnson et al., 2011), spatial information (Bastiaansen et al., 2002), and auditory stimuli (Lim et al., 2015;Obleser et al., 2012;Vogt et al., 1998). In the auditory WM conditions, alpha power was elevated during WM encoding and maintenance, but ongoing oscillations were interrupted by performing either the AT or AS Intervening task. This is consistent with previous work showing that irrelevant visual stimuli interrupt alpha power during retention of both auditory and visual information in WM (Hakim et al., 2020;Mishra et al., 2013). In the visual conditions, alpha power was suppressed relative to baseline during WM encoding—likely because participants needed to attend task-relevant visual stimuli (Foxe & Snyder, 2011;Jensen & Mazaheri, 2010)—but rose toward baseline during WM maintenance. Suppression of alpha power returned after participants performed the AS Intervening task, perhaps due to interference in the visual-biased network, which is geared toward spatial processing. This result should be considered preliminary, however, as differences in the alpha power time courses between the AT and AS Intervening task conditions did not reach statistical significance. Still, the alpha and ERP results both suggest increased interference when both tasks (at least partially) targeted the visual-biased control network due to spatial processing demands. This interference may have been only momentary, as the AS Intervening task did not require mapping stimulus positions into memory. Such ephemeral interference was only detected with the temporal sensitivity of EEG in our paradigm, but it may have meaningful processing consequences in real-world environments with increased competition between sensory inputs, attentional demands, and task-oriented goals.

## 5 Conclusions

In a dual-task paradigm designed to selectively tax auditory- and visual-biased attention and WM networks, we found behavioral, autonomic, and electrophysiological signatures of interference when the two tasks drew upon shared neural control resources. Specifically, when perceptual and working memory tasks were matched in both sensory modality and information domain, behavioral performance was worst, pupil dilations were largest, and ERP amplitudes were suppressed—all indicating increased dual-task interference. These results support resource-specific theories of WM maintenance and indicate that WM control networks are segregated not only on the basis of the sensory information channel, but also based on whether the task requires spatial or temporal information processing. The bidirectionality of interference effects between the two tasks suggests that WM maintenance and perceptual processing relied on shared neural resources in this study, most likely including sensory-biased control networks in the lateral frontal cortex. Future neuroimaging studies should expand this paradigm to explore the dynamics of how LFC control networks support the interplay between WM maintenance and ongoing perceptual processing.

## Data Availability

Data and analysis code are available for download via the Harvard Dataverse (https://doi.org/10.7910/DVN/HBRXVI).
